# Immunopeptidomics Workflow for Isolation and LC-MS/MS Analysis of MHC Class I-Bound Peptides Under Hypoxic Conditions

**DOI:** 10.21769/BioProtoc.5505

**Published:** 2025-11-20

**Authors:** Hala Estephan, Ester M. Hammond, Eleni Adamopoulou

**Affiliations:** 1 Department of Oncology, University of Oxford, Oxford, UK; 2 Centre for Immuno-Oncology, Nuffield Department of Medicine, University of Oxford, Oxford, UK

**Keywords:** MHC class I, Hypoxia, Antigen presentation, Tumor microenvironment, MHC peptide, Mass spectrometry, Immunopeptidomics

## Abstract

Immunopeptidomics enables the identification of peptides presented by major histocompatibility complex (MHC) molecules, offering insights into antigen presentation and immune recognition. Understanding these mechanisms in hypoxic conditions is crucial for deciphering immune responses within the tumor microenvironment. Current immunopeptidomics approaches do not capture hypoxia-induced changes in the repertoire of MHC-presented peptides. This protocol describes the isolation of MHC class I-bound peptides from in vitro hypoxia-treated cells, followed by liquid chromatography-tandem mass spectrometry (LC-MS/MS) analysis. It describes optimized steps for cell lysis, immunoaffinity purification, peptide elution, and MS-compatible preparation under controlled low-oxygen conditions. The method is compatible with various quantitative mass spectrometry approaches and can be adapted to different cell types. This workflow provides a reliable and reproducible approach to studying antigen presentation under hypoxic conditions, thereby enhancing physiological relevance and facilitating deeper immunological insights.

Key features

• Enables isolation of MHC class I-bound peptides from cells cultured under hypoxic conditions.

• Designed for low-input samples and optimized for maintaining cell viability during extended hypoxic exposure.

• Compatible with label-free LC-MS/MS for detailed immunopeptidome analysis.

• Adaptable to all human and murine cell lines commonly used in cancer and immunology research.

## Graphical overview


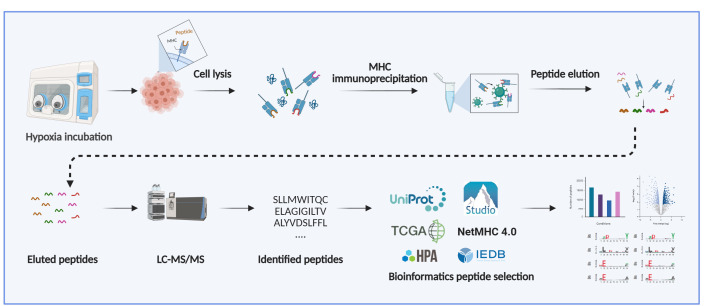


## Background

The presentation of intracellular peptides by major histocompatibility complex (MHC) class-I molecules plays a central role in immune surveillance and the activation of cytotoxic CD8^+^ T lymphocytes [1–3]. Endogenous proteins are continuously degraded in the cytosol into peptide fragments, which are then transported into the endoplasmic reticulum (ER) by the Transporter Associated Protein (TAP) with antigen processing machinery. In the ER, peptides are trimmed to 8-11 amino acids and loaded onto MHC class-I molecules. These peptide-MHC class-I complexes are then transported to the cell surface, where they are recognized by CD8^+^ T cells for signs of infection, malignancy, or other cellular abnormalities [4,5]. The repertoire of the presented peptides by a given group of cells is termed the *immunopeptidome*. Recognition of a non-self or altered-self peptide derived from proteins can trigger targeted elimination of the presenting cell, thus contributing to pathogen clearance and tumor surveillance [6].

The ability to accurately identify MHC class I-bound peptides is essential for characterizing T-cell immunity at the level of individual tumor-specific peptides for developing more effective immunotherapies, including vaccines [7–9]. Mass spectrometry-based immunopeptidomics is a powerful methodology for characterizing the cancer cell immunopeptidome, including the discovery of tumor-specific antigens. [7,10]. However, the analysis of naturally presented MHC class I-bound peptides remains particularly challenging due to their low abundance, lack of standardized proteolytic cleavage, and high sequence similarity driven by MHC-binding preferences [11–13]. Additional challenges include optimizing sample preparation for diverse cell types, preventing loss of low-abundance peptides, addressing mass spectrometry biases, and adapting bioinformatic pipelines to reliably identify non-tryptic peptides.

The protocol presented here provides a reproducible workflow designed to facilitate robust immunopeptidomics analysis. Recent advances in mass spectrometry sensitivity, immunoaffinity purification protocols, and bioinformatic pipelines have significantly improved the depth and resolution of immunopeptidome analyses [14]. Importantly, the immunopeptidome reflects dynamic changes in cellular states, including those induced by stress, inflammation, or metabolic shifts, making it a rich source of potential diagnostic and therapeutic targets [15].

This growing interest in antigen presentation has led to the development of protocols capable of better capturing the full repertoire of peptides displayed by MHC class I and II under various physiological and pathological conditions, including hypoxia, a common feature of the tumor microenvironment [16,17]. Mapping these context-dependent changes is critical for identifying tumor-specific antigens, understanding immune escape mechanisms, and improving the efficacy of T cell-based therapies [18–20].

## Materials and reagents

**Biological materials**

Cancer cell lines are grown in culture using adequate media and maintained in humidified incubators at 37 °C with 5% CO_2_. **Caution:** Routine validation of cell line identity (STR profiling) and mycoplasma testing is strongly recommended.

**Reagents**

**Critical:** Use only LC-MS-grade water, solvents, and high-purity reagents in all procedures. All glassware must be thoroughly cleaned with LC-MS-grade solvents (e.g., methanol) and LC-MS-grade water before use. Both glassware and plasticware can release polymeric contaminants, plasticizers, or detergent residues that interfere with peptide detection in LC-MS/MS analyses. To minimize such contamination, employ ultraclean glassware and high-quality, low-extractable plasticware. We strongly recommend verifying all tubes and consumables for leachable compounds before use. Where indicated, prepare solutions fresh before each experiment and discard any remaining solutions after no more than one week of storage. Use glass bottles for solution storage to reduce the risk of polymeric contaminants that can accumulate over time.

1. Formic acid (LC-MS ultra, >98% purity) (Sigma-Aldrich, catalog number: 10596814)

2. Acetic acid (glacial; HPLC-grade) (Sigma-Aldrich, catalog number: 10365020)

3. Acetonitrile (ACN) (LC-MS-grade) (Thermo Fisher Scientific, catalog number: 1.00029.2500)

4. Water (LC-MS-grade) (Thermo Fisher Scientific, catalog number: 1.15333.2500)

5. Trifluoroacetic acid (TFA) (LC-MS-grade) [Sigma-Aldrich, catalog number: 10723857 (A116-50)]

**Caution:** TFA is corrosive; wear appropriate personal protective equipment (PPE) accordingly. Buffers should be prepared in a fume hood due to the risk of toxic emissions.

6. Dimethyl pimelimidate dihydrochloride (Sigma-Aldrich, catalog number: D8388-250MG)

7. Complete protease inhibitor (Roche, catalog number: 11836145001)

8. IGEPAL^®^ CA 630 (Abcam, catalog number: ab285400)

9. 0.5 M EDTA, pH 8 (Sigma-Aldrich, catalog number: 46-034-CI)

10. Protein A resin (Abcam, catalog number: ab270308-200mL)

11. Protein G resin (Abcam, catalog number: ab270309-100mL)

**Critical:** Check the isotype of the monoclonal antibody (mAb) to be used, as protein G resin may be more appropriate (e.g., for the IgG1 isotype). Refer to Table 1 for detailed information on the most commonly used anti-human antibodies.

**Table 1.** Commonly used anti-human antibodies for immunoaffinity capture of MHC-peptide complexes

Hybridoma	Specificity	Isotype	Sepharose	ATCC
W6/32	HLA -A, -B, -C	IgG2a	A/G	HB-95
MA2.1	HLA-A2, -B17	IgG1	G	HB-54
BB7.2	HLA-A2, -Aw69	IgG2b	A/G	HB-82
GAP A3	HLA-A3	IgG2a	A/G	HB-122
TM1	HLA-A2, -A28, -Bw4, -B27, -B44, -Cw2	IgM	A	HB-169
ME1	HLA-B7, -Bw22, -B27, -B14, -Bw46	IgG1	G	HB-119
DT9	HLA-C (HLA-E)	IgG2b	A/G	-
3D12	HLA-E	IgG1k	G	Available under a licensing agreement from Fred Hutchinson Cancer Center

12. PBS (1×, sterile) (Sigma-Aldrich, catalog number: J61196.AP)

13. H_3_BO_3_ (boric acid), pH 5.1 (25 °C, 1.8 g/L) (Sigma-Aldrich, catalog number: B6768-500G)

14. Potassium chloride (KCl) (Sigma-Aldrich, catalog number: P9541-500G)

15. Sodium hydroxide (NaOH) (Sigma-Aldrich, catalog number: S8045-500G)

16. Sodium chloride (NaCl) (Sigma-Aldrich, catalog number: 31434-1KG-M)

17. Tris, ultra-pure grade (Sigma-Aldrich, catalog number: T6066-1KG)

18. pan MHC I antibody clone W6/32 (produced in house, hybridoma cells HB-95)

19. Dulbecco’s modified Eagle medium (Gibco, catalog number: 10313021)

20. Fetal bovine serum (Sigma-Aldrich, catalog number: F7524-500ML)

21. Penicillin-streptomycin (Sigma-Aldrich, catalog number: 15070063)

22. 0.25% trypsin-EDTA (Sigma-Aldrich, catalog number: T3924)

23. Purified mouse anti-human GRP78 (BD Biosciences, catalog number: 610978)

24. Purified mouse and-human HIF-1α (BD Biosciences, catalog number: 610959)

25. Goat anti-mouse IgG (H+L) (Licor Biosciences, catalog number: IRDye800RD)

26. LICOR blocking buffer (Licor Biosciences, catalog number: 927-60003)

27. 10 well, 50 μL gels (4%–20%) (Bio-Rad, catalog number: 4561094)

28. 12 well, 20 μL gels (4%–20%) (Bio-Rad, catalog number: 4561095)

29. Turbo Transfer kit (Bio-Rad, catalog number: 1704270)

30. Western Blot protein ladder (Bio-Rad, catalog number: 1610374)

**Solutions**

1. Boric acid-KCl stock solution (see Recipes)

2. Borate wash buffer (see Recipes)

3. Dimethyl pimelimidate (DMP) cross-linker (see Recipes)

4. Termination buffer (see Recipes)

5. 2× Lysis buffer (see Recipes)

6. Wash buffer 1 (see Recipes)

7. Wash buffer 2 (see Recipes)

8. Wash buffer 3 (see Recipes)

9. Wash buffer 4 (see Recipes)

10. 10% (v/v) acetic acid (see Recipes)

11. 1 M NaOH (see Recipes)

12. 0.1 M NaOH (see Recipes)

13. 1 M Tris, pH 8.0 (see Recipes)

14. 20% ACN 0.1% TFA (see Recipes)

15. 80% ACN 0.1% TFA (see Recipes)

16. 50% ACN 0.1% TFA (see Recipes)

17. 1% ACN 0.1% TFA (see Recipes)

18. 0.1% TFA (see Recipes)

19. Mass spectrometry loading buffer (see Recipes)

**Recipes**

**1. Boric acid-KCl stock solution**

**Table:** 

Reagent	Final concentration	Quantity or volume
Boric acid	0.1 M	0.62 g
KCl	0.1 M	0.75 g
LC-MS-grade water	-	100 mL

**2. Borate wash buffer**

**Table:** 

Reagent	Final concentration	Quantity or volume
NaOH	0.1 M	4 mL
Boric acid-KCl	0.1 M	50 mL
LC-MS-grade water	-	100 mL

**3. Dimethyl pimelimidate (DMP) cross-linker**

Prepare 40 mM DMP by dissolving 250 mg of DMP in 8 mL of borate wash buffer. Adjust the pH to 9.0 with 1 M NaOH. Top up to 25 mL with borate wash buffer. Prepare this solution fresh, immediately before use.

**Critical:** DMP powder can be stored at -20 °C; however, prolonged storage or repeated freeze–thaw cycles may reduce its cross-linking efficiency. To avoid stability issues, we recommend using single-use 250 mg vials, which are sufficient to crosslink up to 20 mg of antibody to 2 mL of resin.

**4. Termination buffer**

**Table:** 

Reagent	Final concentration	Quantity or volume
Tris pH 8.0	0.2 M	20 mL from 1 M Tris stock solution
LC-MS-grade water	-	80 mL

**5. 2× Lysis buffer**

**Table:** 

Reagent	Final concentration	Quantity or volume
Tris pH 8.0	100 mM	10 mL from 1 M Tris stock
NaCl	300 mM	20 mL from 1.5 M NaCl stock
IGEPAL	1%	10 mL from 10% IGEPAL
LC-MS-grade water	-	60 mL

**Critical:** 2× lysis buffer can be stored at 4 °C for 1–2 weeks. For optimal reproducibility, mix thoroughly before use and prepare fresh buffer if longer storage is required.

Add the protease inhibitor cocktail before use; dissolve one tablet in 10 mL of lysis buffer. Add phosphatase inhibitor if required (PhosStop, Roche) before use (1 tablet/10 mL of lysis buffer). Keep the lysis buffer cold.

**6. Wash buffer 1**

**Table:** 

Reagent	Final concentration	Quantity or volume
Tris pH 8.0	50 mM	5 mL from 1 M Tris stock
NaCl	150 mM	10 mL from 1.5 M NaCl stock
EDTA	5 mM	1 mL from 0.5 M EDTA
LC-MS-grade water	-	84 mL

**7. Wash buffer 2**

**Table:** 

Reagent	Final concentration	Quantity or volume
Tris pH 8.0	50 mM	5 mL from 1 M Tris stock
NaCl	150 mM	10 mL from 1.5 M NaCl stock
LC-MS-grade water	-	85 mL

**8. Wash buffer 3**

**Table:** 

Reagent	Final concentration	Quantity or volume
Tris pH 8.0	50 mM	5 mL from 1 M Tris stock
NaCl	450 mM	30 mL from 1.5 M NaCl stock
LC-MS-grade water	-	65 mL

**9. Wash buffer 4**

**Table:** 

Reagent	Final concentration	Quantity or volume
Tris pH 8.0	50 mM	5 mL from 1 M Tris stock
LC-MS-grade water	-	95 mL

**10. 10% (v/v) acetic acid**

**Table:** 

Reagent	Final concentration	Quantity or volume
Glacial acetic acid	10%	25 mL
LC-MS-grade water	-	225 mL

**11. 1 M NaOH**

**Table:** 

Reagent	Final concentration	Quantity or volume
NaOH (Mr = 40 g/mol)	1 M	4 g
LC-MS-grade water	-	100 mL

**12. 0.1 M NaOH**

**Table:** 

Reagent	Final concentration	Quantity or volume
NaOH (Mr = 40 g/mol)	0.1 M	0.4 g
LC-MS-grade water	-	100 mL

**13. 1 M Tris, pH 8.0**

**Table:** 

Reagent	Final concentration	Quantity or volume
Tris (Mr = 121.14 g/mol)	1 M	121.14 g
LC-MS-grade water	-	800 mL

Adjust pH with HCl; approximately 42 mL of HCl is needed to achieve pH 8.0.

**14. 20% ACN 0.1% TFA**

**Table:** 

Reagent	Final concentration	Quantity or volume
Acetonitrile	20%	10 mL
TFA	0.1%	50 μL
LC-MS-grade water	-	39.95 mL

**15. 80% ACN 0.1% TFA**

**Table:** 

Reagent	Final concentration	Quantity or volume
Acetonitrile	80%	40 mL
TFA	0.1%	50 μL
LC-MS-grade water	-	mL

**16. 50% ACN 0.1% TFA**

**Table:** 

Reagent	Final concentration	Quantity or volume
Acetonitrile	50%	25 mL
TFA	0.1%	50 μL
LC-MS-grade water	-	mL

**17. 1% ACN 0.1% TFA**

**Table:** 

Reagent	Final concentration	Quantity or volume
Acetonitrile	1%	500 μL
TFA	0.1%	50 μL
LC-MS-grade water	-	49.45 mL

**18. 0.1% TFA**

**Table:** 

Reagent	Final concentration	Quantity or volume
TFA	0.1%	50 μL
LC-MS-grade water	-	49.45 mL

**19. Mass spectrometry loading buffer**

**Table:** 

Reagent	Final concentration	Quantity or volume
Acetonitrile	1%	500 μL
Formic acid	0.1%	50 μL
LC-MS-grade water	-	49.45 mL

**Laboratory supplies**

1. Petri dish, Duroplan, borosilicate glass, diameter: 143 mm, height: 30 mm (VWR International, catalog number: 391-0860)

2. 150 mm TC dish, sterile, non-pyrogenic (Fisher Scientific, catalog number: 11804125)

3. TC cell scraper (Sarstedt, catalog number: 83.3951)

4. 10 μL filter pipette tips, hinged, extended length, graduated (Appleton Woods, catalog number: ACA619)

5. 20 μL filter pipette tips, hinged, extended length, graduated (Appleton Woods, catalog number: ACA626)

6. 200 μL filter pipette tips, hinged, extended length, graduated (Appleton Woods, catalog number: ACA659)

7. 1,250 μL filter pipette tips, hinged, extended length, graduated (Appleton Woods, catalog number: ACA668)

8. Protein LoBind tube 1.5 mL, PCR clean (Fisher Scientific, catalog number: 10708704)

9. Protein LoBind tube 2 mL, PCR clean (Fisher Scientific, catalog number: 10718874)

10. Econo-Column^®^ chromatography columns (Bio-Rad, catalog number: 737-1012)

11. Ultrafree^®^-MC centrifugal filter units (Merck, catalog number: UFC3LCCNB-HMT)

12. Falcon 15 mL centrifuge tubes (Fisher Scientific, catalog number: 352097)

13. Falcon 50 mL centrifuge tubes (Fisher Scientific, catalog number: 352098)

14. Pierce^TM^ centrifuge columns (Thermo Fisher Scientific, catalog number: 89868)

15. Pierce^TM^ C18 spin tips, 96 tips (Thermo Fisher Scientific, catalog number: 84850)

16. Certified kits, screw thread vials, 12 × 32 mm, 9 mm thread, unassembled (Merck, catalog number: 29385-U)

17. pH strips (Fisher Scientific, catalog number: 10642751)

18. Pierce^TM^ BCA Protein Assay kit (Thermo Scientific, catalog number: 23225)

19. Anaerobic indicator strips (Fisher Scientific, catalog number: BR0055B)

## Equipment

1. Calibrated pH meter

2. Benchtop centrifuge

3. Chromatography column (Bio-Rad, model: Econo-Column^®^)

4. Shaker (IKA, model: VX 2E.n)

5. Orbital rotator

6. Tube rotator (Camlab, catalog number: 51901-26)

7. Ultrasonic USC-TH sonicator bath

8. Hypoxia chamber (Don Whitley Scientific)

9. Racks (Thermo Fischer)

10. Freezer (-20 °C and -80 °C)

11. Refrigerator (2–8 °C)

12. LC-Mass Spectrometer

13. Trans-Blot Turbo system (Bio-Rad)

14. Buffer tank and lid (Bio-Rad)

15. Gel holder cassette (Bio-Rad)

16. OxyLite probe (Oxford Optronix)

## Software and datasets

High-resolution mass spectrometry generates large datasets that require computational processing to identify peptide sequences from fragmentation spectra. Common search algorithms, including MASCOT, SEQUEST, MaxQuant, and Protein Pilot, perform this task by searching spectra against fragmentation spectra databases and de novo peptide sequencing. In immunopeptidomics, where MHC-bound peptides are not produced by enzymatic digestion and therefore do not follow trypsin-specific cleavage rules [cleavage after arginine (R) and lysine (K)], hybrid approaches are strongly recommended. Computational tools like PEAKS combine database searching with de novo sequencing, first generating candidate peptide sequences through de novo analysis to guide the database search. This strategy enhances both search efficiency and identification accuracy, which is critical given the non-tryptic nature of MHC-associated peptides. In this protocol, we used PEAKS Studio v10.0 (Bioinformatics Solutions Inc.), as its integration of de novo sequencing with database searching provides improved identification of non-tryptic peptides compared to conventional search engines focused on tryptic peptides.

*Note: PEAKS (commercial license required;* *https://www.bioinfor.com/peaks/* *), NetMHC (freely available;* *https://services.healthtech.dtu.dk/services/NetMHCpan-4.1/* *), IEDB (freely available;* *https://www.iedb.org/* *), WebLogo (freely available;* *http://weblogo.threeplusone.com/* *), FragPipe (freely available;* *https://fragpipe.nesvilab.org/* *), and MHCquant 2 (freely available;* *https://www.mhquant.org/* *) are commonly used in these analyses.*

## Procedure

**A. Generation of cell lysates**

*Note: The amount of starting material required depends on the LC-MS/MS platform used, the desired analytical depth, and the level of HLA expression in the cell sample. For instance, cancer cell lines cultured in vitro are among the simplest sample types for MHC-peptide isolation, typically requiring between 5 × 10^7^ and 1 × 10^9^ cells per preparation.*

1. Culture cells in the appropriate media at 37 °C in a humidified incubator containing 5% CO_2_, until approximately 80% confluency is reached.

2. For hypoxic samples:

a. Incubate the cells at the required level of oxygen for the desired time before harvesting. Perform all harvesting steps inside the hypoxic chamber to prevent reoxygenation.

b. Pre-equilibrate PBS and collection tubes in the hypoxia chamber overnight to minimize oxygen exposure.

c. Remove the culture medium and wash cells twice with PBS.

d. Detach adherent cells using a cell scraper in PBS.

e. Transfer the suspension to 15 or 50 mL conical tubes and centrifuge at 500× *g* for 5 min at 4 °C. Return tubes to the chamber to discard the supernatant.

f. Resuspend the pellet with PBS and centrifuge.

g. Carefully remove and discard the supernatant.

h. Use pelleted cells directly for HLA-I profiling or snap freeze in liquid nitrogen for future use.

*Note: Subsequent steps of the protocol can be performed outside the hypoxia chamber.*

**Critical:** When conducting experiments under very low oxygen conditions, use glass dishes instead of plastic ones, as glass helps minimize oxygen retention that could affect the results. Ensure that hypoxic incubation is performed in a hypoxia chamber rather than a standard incubator set to low oxygen. Using a dedicated hypoxia chamber maintains stable low-oxygen conditions throughout incubation and prevents reoxygenation artifacts. The absence of traces of oxygen can be periodically verified by using anaerobic indicator strips. To further verify oxygen levels, an OxyLite probe can be used to determine oxygen levels in the media surrounding cells exposed to hypoxia.

**Figure 1.**
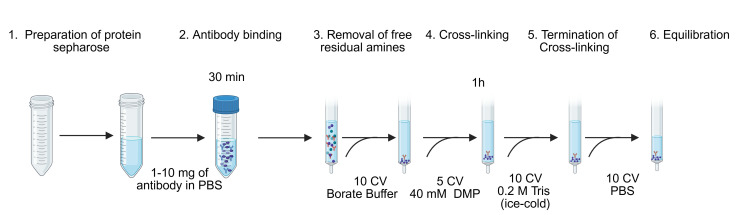
Overview of immunopeptidomics data analysis for HCT116 cells exposed to normoxia or hypoxia for 24 h. (A) Representative western blot of HT29 cells exposed to hypoxic conditions (<0.1% O_2_) for the indicated time points. (B) Total number of major histocompatibility complex (MHC) I peptides presented on HCT116 cells in normoxia and hypoxia (<0.1% O_2_). (C) Unique number of MHC I peptides in normoxia vs. hypoxia after 24 h. (D) Allele binding distribution of MHC I peptides. (E) Length frequency distribution of MHC I peptides. (F) Seqlogo comparison of MHC binding motifs in normoxia and hypoxia for three different MHC I subtypes. Peptides were assessed using NetMHCPan 4.1. Data represent mean **±** SEM from three biological replicates. Statistical significance was determined using an unpaired Student’s t-test.

**Critical:** For hypoxia-treated samples, it is essential to confirm that cells were indeed hypoxic at the time of harvest. Include an additional dish cultured and collected in parallel and validate hypoxia by measuring HIF-1α protein levels using western blot analysis. For experiments performed under more severe hypoxic conditions (<0.1% O_2_), activation of the unfolded protein response (UPR) can be confirmed by assessing increased GRP78 expression, which serves as an additional marker of cellular adaptation to low oxygen [21]. A representative western blot illustrating typical results is provided in Figure 1A.

3. For normoxic samples:

a. Transfer the cell suspension to 15 or 50 mL conical tubes and pellet cells by centrifugation at 500× *g* for 5 min at 4 °C.

b. Carefully remove and discard the supernatant.

c. Resuspend cells and wash twice in cold PBS.

d. Carefully remove and discard the supernatant.

e. Use pelleted cells directly for HLA-I profiling or snap freeze in liquid nitrogen for future use.

f. Dry cell pellets can be stored at -80 °C up to 6 months.

**B. Preparation of cross-linked beads**

**Figure 2.**
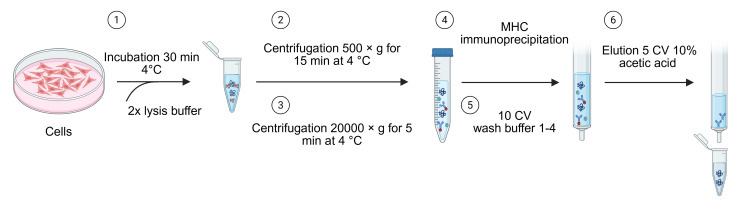
Generation of MHC immunoaffinity column. The first six steps of the procedure are shown. CV, column volume; DMP, dimethyl pimelimidate dihydrochloride.

*Note: Refer to Figure 2 for a schematic overview of the different steps.*

1. Wash the column to remove polymerics and other contaminants: Incubate the column with 10% acetic acid for at least 30 min. Thoroughly wash with PBS and verify that the pH is neutral before proceeding.

2. Prepare protein A Sepharose beads: Resuspend the beads gently by slow inversion or rolling; avoid vigorous shaking. Transfer the required volume of protein A Sepharose [supplied as a 50% (w/v) slurry in 20% (v/v) ethanol] into a 50 mL conical tube. Fill the tube with PBS, then centrifuge at 100× *g* for 1 min to pellet the beads. Carefully discard the ethanol-containing supernatant.

**Critical:** Do not pipette up and down when resuspending, as this can damage the beads. The volume of protein A Sepharose beads utilized is proportional to the quantity of antibody required for each experiment; in general, 50 μL of dry beads is used per 1 mg of antibody.

3. Antibody coupling: Prepare the MHC I monoclonal antibody (clone W6/32) at a concentration of 0.5–1 mg/mL in PBS. Add the antibody to the washed protein A Sepharose and incubate for 30 min on an orbital rotator at ~11 rpm at room temperature (RT). Alternatively, incubate for 1–4 h (minimum 1 h) on an orbital rotator at 4 °C.

**Critical:** Before cross-linking, confirm the quality and specificity of the antibody batch by assessing its binding properties using flow cytometry.

4. Remove residual free amines: Transfer the antibody-bound resin back into the column. Wash the resin with 10 column volumes (CV) of borate wash buffer, allowing the buffer to drain fully.

**Critical:** Removing residual primary amines is essential, as they can react with the cross-linker and reduce efficiency. Do not let the column dry during this step.

5. Cross-link the antibody: Add 10 CV of freshly prepared DMP cross-linking solution to the column at RT and allow it to drain until the meniscus is just above the resin bed. Seal the bottom of the column and incubate for 30 min at RT.

6. Terminate cross-linking: Wash the beads with 10 CV of ice-cold 0.2 M Tris, pH 8.0, to quench the cross-linking reaction.

7. Neutralize and wash: Wash the beads with 10 CV of the termination buffer, 50 mM Tris, pH 8.0. Confirm that the pH of the resin is neutral before storing or using it further.

8. Recover the immuno-resin: Using a stripette (10 mL), gently resuspend the beads and transfer them into a clean container with an appropriate volume of 50 mM Tris, pH 8.0.

**Pause point:** At this stage, the cross-linked immuno-resin can be stored at 4 °C for up to 1 month.

**C. Generation of cell lysate and MHC immunoaffinity purification**

**Figure 3.**
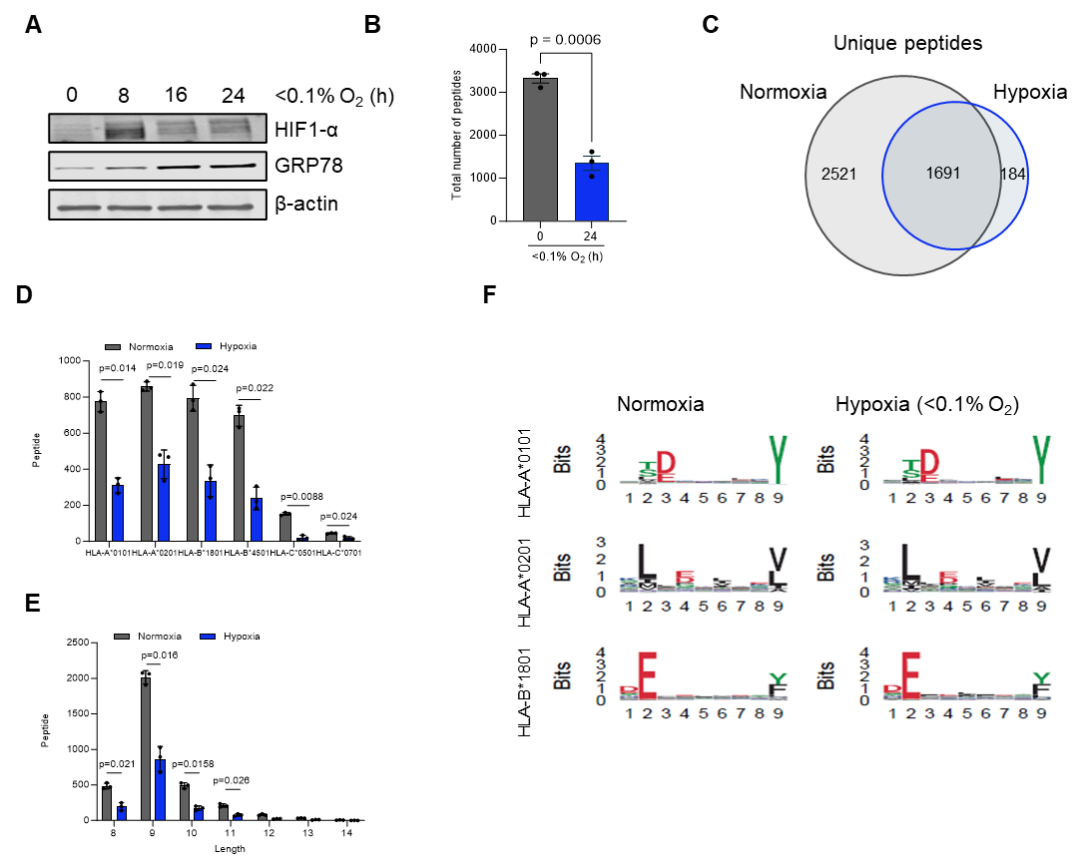
Main steps of the procedure: Generation of cell lysate, immunopurification, and elution of MHC–peptide complexes

*Note: Refer to Figure 3 for a schematic showing the different steps.*

1. Thaw samples on ice to minimize cell lysis-induced DNA release.

2. Add approximately 0.5 mL of 2× ice-cold lysis buffer to each sample. Mix gently by slow pipetting using low-retention tips until the lysate is homogeneous. Adjust the volume with additional lysis buffer if needed to ensure complete resuspension.

3. Incubate the lysate on an orbital rotator at 4 °C for 30 min to solubilize MHC complexes.

*Note: Do not exceed 30 min to preserve complex integrity.*

4. Remove nuclei: Centrifuge the lysate at 500× *g* for 15 min at 4 °C. Carefully collect the supernatant and transfer it into a fresh tube. Freeze the nuclei pellet at -80 °C for potential genomic DNA extraction and downstream WGS analysis.

5. Clarify the lysate: Transfer the supernatant into a pre-chilled and pre-labeled 2 mL LoBind Eppendorf tube. Centrifuge at 20,000× *g* for 45 min at 4 °C to remove insoluble debris.

**Optional:** Perform a BCA protein assay to quantify total protein content. Normalize all samples to the lowest measured protein concentration by adding an appropriate volume of 50 mM Tris, pH 8.0, to adjust the final lysis buffer concentration to 1×.

6. Prepare the immunoaffinity resin: Aliquot the required volume of cross-linked immuno-resin into 2 mL LoBind Eppendorf tubes.

7. Immunoprecipitation: Add the clarified lysate to the cross-linked beads (recommended ratio: 0.5–2 mL lysate per 20 μL dry beads). Incubate overnight (12–16 h) at 4 °C on an orbital rotator or tube rotator at ~10–15 rpm to ensure gentle mixing and efficient capture of MHC complexes. Use a minimum lysate volume of 0.5 mL to fully suspend the resin; for volumes exceeding 2 mL, divide the lysate across multiple tubes to maintain optimal bead-lysate contact.

**D. Removal of residual contaminants and elution of MHC-peptide complexes**

**Critical:** For optimal performance, prepare the wash buffers immediately before use. Maintain all buffers and reagents cold during the procedure.

1. Prepare the columns: Incubate the columns with 10% acetic acid for 30 min to remove residual contaminants. Wash thoroughly with PBS.

**Caution:** Verify that the pH is neutral using pH test strips (0–14) before loading samples.

2. Load the lysate: Transfer the clarified lysate onto the columns containing the immuno-resin. Collect the flowthrough in pre-labeled Eppendorf tubes and store at -80 °C for downstream analyses, including MHC II-peptide complex immunoprecipitation.

3. Wash the beads: Sequentially wash the resin with at least 10 CVs of the following buffers:

a. Wash buffer 1: Low-salt chelating buffer; removes residual detergents and weakly bound contaminants; EDTA chelates divalent cations to reduce nonspecific binding.

b. Wash buffer 2: Low-salt buffer; removes residual detergents and nonspecifically interacting proteins.

c. Wash buffer 3: High-salt buffer; removes strongly nonspecifically bound proteins due to increased ionic strength.

d. Wash buffer 4: Very low-salt buffer; final wash to remove excess salt and prevent crystal formation before elution.

**Caution:** Do not allow the immune resin to dry.

4. Elute the MHC-peptide complexes: Add 5 CVs of 10% acetic acid to the column. Collect the eluate into LoBind 2 mL Eppendorf tubes. Use a syringe plunger to gently push out any remaining liquid to maximize recovery.

5. Proceed to peptide separation: Continue with the separation of peptides from the MHC-peptide complexes.

**E. Separation of peptides from MHC-peptide complexes**

1. Pre-condition filter units: For each sample, fill an ultra-free MC-PLHCC centrifugal filter unit with 500 μL of 10% acetic acid.

2. Centrifuge at 15,000× *g* for 45–60 min at 4 °C to remove residual contaminants.

3. Remove the filter from the collection tube, discard the flowthrough, and rinse the bottom of the filter by gently pipetting up and down with 500 μL of 10% acetic acid to ensure complete removal of residual liquid.

4. Return the filter insert to the clean collection tube.

5. Load samples: Add the MHC I complex eluates to the pre-conditioned, pre-labeled filter units.

6. Centrifuge at 15,000× *g* for ~1 h at 4 °C (for a ~500 μL sample volume).

*Note: If the sample volume exceeds the filter capacity, spin in multiple batches; keep remaining sample aliquots on ice between spins. Collect the filtrate in pre-labeled low-binding Eppendorf tubes.*

7. Transfer the collected filtrates to fresh, pre-labeled Eppendorf tubes.

8. Peptide recovery wash: Add 50 μL of 20% ACN 0.1% TFA directly onto the filter membrane.

**Critical:** The peptide recovery wash with 20% ACN 0.1% TFA is required to ensure complete recovery of peptides remaining on the filter membrane. Always combine this wash with the main filtrate for downstream LC-MS/MS analysis to maximize peptide yield, especially for low-abundance MHC-bound peptides.

9. Centrifuge again at 15,000× *g* for 30 min at 4 °C to recover any residual peptides.

**Optional:** Spike-in control for peptide recovery: To monitor recovery of low-abundance peptides, add a defined amount of a synthetic or isotopically labeled spike-in peptide to the cell lysate before immunoaffinity purification. Analyze the spike-in peptide alongside endogenous peptides during LC-MS/MS. This enables users to assess recovery efficiency and identify potential issues throughout the workflow.

10. Pool the eluate with the main filtrate collected in step E6.

11. Dry the combined peptide eluates using a Speed-Vac concentrator.

**Pause point:** Dried peptides can be stored at -20 °C until further LC-MS analysis.

**F. Desalting with Pierce^TM^ C18 spin tips**

**Critical:** For best results, prepare reagents freshly before use. Discard unused reagents after one week of storage.

1. Sample resuspension: Reconstitute dried peptide samples in 50 μL of 1% ACN 0.1% TFA. Confirm that the solution pH is <4 using pH test strips.

**Critical:** Avoid pipetting up and down to minimize peptide loss and prevent bubble formation. Do not vortex, as this can cause foaming and reduce recovery of low-abundance peptides.

2. Place tubes on an orbital shaker and mix at 1,000× *g* for 10–15 min (IKA VX 2E.n).

3. Sonicate samples for 3–5 min in a water bath sonicator without temperature control.

4. Clarify the resuspended samples by centrifugation at 20,000× *g* (maximum speed) for 10 min at 4 °C.

5. Insert the Pierce^TM^ C18 spin tip into the spin adapter seated inside a clean 2 mL microcentrifuge tube.

6. Tip conditioning: Add 20 μL of 80% ACN 0.1% TFA to the spin tip. Centrifuge at 1,000× *g* for 1 min to wet the C18 resin.

7. Equilibration: Add 20 μL of 0.1% TFA to the tip and centrifuge at 1,000× *g* for 1 min. Repeat this equilibration step once more.

8. Discard the collection tubes containing the flowthrough.

9. Sample loading: Carefully apply 20–50 μL of clarified peptide solution onto the equilibrated spin tip, using the original sample Eppendorf tube as the collection vessel. Centrifuge at 1,000× *g* for 1 min.

**Critical:** Confirm visually that the entire sample has passed through the tip. For larger volumes or slower flow rates, extend centrifugation time to 2–3 min, if necessary, but do not increase the centrifuge speed.

10. Add 20 μL of 0.1% TFA to the tip and centrifuge at 1,000× *g* for 1 min. Repeat this wash once more to remove residual contaminants.

11. Transfer the spin tip and adapter to a new, pre-labeled Eppendorf tube. Discard the previous collection tube.

12. Add 20 μL of 50% ACN 0.1% TFA to the tip. Centrifuge at 1,000× *g* for 1 min to elute peptides. Repeat this elution step once more to maximize peptide recovery.

13. Dry the pooled eluates in a Speed-Vac concentrator.

**Critical:** Avoid drying completely to prevent loss of hydrophilic peptides by adsorption to the tube walls.

14. Proceed immediately with LC-MS/MS sample preparation, or store partially dried samples at -80 °C until analysis.

**G. Preparation of samples for LC-MS/MS**

1. Sample resuspension: Add 15–25 μL of 0.1% formic acid to each dried peptide sample to fully resuspend the peptides.

2. Place the tubes in a sonicating water bath for 5 min to help release peptides.

**Critical:** Ensure complete resuspension of peptides to avoid sample loss.

3. Centrifuge the samples at 13,000× *g* for 10 min at 4 °C to pellet any insoluble particulates. Carefully transfer the clear supernatant into clean autosampler vials, avoiding bubble formation to ensure the sample settles at the bottom.

**Critical:** Failure to remove particulates can clog the downstream nano-LC system. Use low-bind vials to prevent peptide adhesion.

4. Injection and analysis: Load the prepared samples onto the mass spectrometer and run using an optimized LC gradient. Suggested settings for Thermo Fisher Q-Exactive-HF are detailed in Table 2.

**Critical:** Data acquisition parameters, such as inclusion of singly charged peptides, number of ions selected per cycle, and accumulation time, should be carefully optimized to ensure the collection of high-quality, information-rich MS/MS spectra.

Use high-mass-accuracy analyzers (e.g., Orbitrap or TOF instruments) instead of lower-accuracy systems like ion traps to increase confidence in peptide identifications, particularly when combining de novo sequencing with database searching. Optimize acquisition parameters by setting high ion targets and longer accumulation times (e.g., 100–120 ms) to enhance MS/MS spectral quality and increase spectral yield (up to ~30%–40%). On Orbitrap systems, a high ion target prevents limiting the maximum injection time. Including singly charged precursors can further boost identifications, especially for alleles producing peptides with few basic residues, but these should be fragmented at higher collision energy and lower priority to reduce co-isolation of contaminants.

**Table 2.** Instrument-specific settings for the Thermo Scientific Q-Exactive HF mass spectrometer

Mass spectrometry parameters	Values
Spray voltage Capillary temperature	1,700–2,200 V 275 °C
Full-scan MS range	300–1,800 m/z
MS1 resolution (m/z 200)	70,000
Ion target	5 × 10^5^
Maximum injection time	120 ms
Precursors selected for MS/MS	Top 20 per cycle
Precursor charge state	2–4+
Higher-energy collisional dissociation	27%
MS2 accumulation time	120 ms
MS/MS resolution (Orbitrap)	35,000
Target	1 × 10^5^
Isolation width	1.2 Da
Underfill ratio	1%
Dynamic exclusion	15 s

## Data analysis

1. Database search: Analyze the raw MS/MS data using your preferred search engine and protein database. In the workflow presented here, PEAKS Studio v10.0 was used with parameters optimized for the identification of MHC class-I peptides. Searches were performed with no enzyme specificity and restricted to 8–12 amino acid-long peptides, reflecting typical MHC I ligands. Mass tolerances were set to 10 ppm for precursor ions and 0.02 Da for fragment ions. Variable modifications included oxidation (M), deamidation (N/Q), phosphorylation (S/T/Y), and cysteinylation (C). Peptide identifications were filtered to a 1% FDR (false discovery rate) at the peptide level, and searches were conducted against the UniProt human reference proteome supplemented with common contaminants (Figure 1B, C).

*Note: Most search engines are protein-centric and may penalize proteins represented by only a single peptide; this is a common feature in immunopeptidomic datasets, where many MHC I peptides appear as singletons [22].*

2. Curation and motif analysis: Inspect the list of identified peptides. Evaluate peptide lengths, motifs, and predicted MHC binding to confirm data quality. Tools such as the Immune Epitope Database (IEDB; http://www.iedb.org/) and NetMHC 4.0 (https://services.healthtech.dtu.dk/services/NetMHC-4.1/) can be used to predict binding affinity and assign peptides to their respective HLA alleles [23] (Figure 1D).

3. (Optional) Length distribution: Plot the distribution of peptide lengths (typically 8–12 mers for MHC class I) as a histogram or frequency plot to assess whether the observed peptide repertoire matches expected MHC binding characteristics (Figure 1E).

4. Contaminant removal: Filter out known contaminant peptides, including proteolytic fragments, carryover peptides from pre-columns, buffers, or other laboratory sources to ensure reliable peptide identifications and biological interpretations. Build and maintain a curated list of experimentally identified contaminants from multiple negative controls across MS runs to support this step.

5. Reference comparison using the HLA Ligand Atlas: Cross-reference the identified peptide repertoire against the HLA Ligand Atlas, a large-scale reference database of naturally presented HLA ligands across multiple benign human tissues (PXD019643). This comparison helps annotate peptides as previously observed in non-malignant contexts, deprioritize broadly presented peptides that may represent background or self-antigens, and highlight candidate tumor-enriched ligands. The Atlas can also be used for comparative motif analysis and, when appropriate, for building spectral libraries to support DIA-based workflows.

6. Visualization: Generate sequence logos to illustrate conserved motifs within the identified peptides. This can be done using online tools such as WebLogo 3 (http://weblogo.threeplusone.com) [24] or Seq2Logo (https://services.healthtech.dtu.dk/services/Seq2Logo-2.0/) [25].

7. Clustering analysis: Use unsupervised clustering algorithms, such as the Gibbs clustering method [26] or other clustering tools [27], to group peptides sharing similar binding motifs. Clustering can help refine allele assignments and assist in identifying and removing nonspecifically bound or contaminant peptides (Figure 1F).

## Validation of protocol

This protocol has been used and validated in the following research article:

• Estephan et al. [28]. Hypoxia promotes tumor immune evasion by suppressing MHC-I expression and antigen presentation. *EMBO Journal* (Figures 5B–E and 6 and Supplementary Figures 6, 7, and 9).

## General notes and troubleshooting

**Troubleshooting**

**Problem 1:** Low yield of MHC I-peptide complexes.

Possible causes: Antibody poor binding or failed cross-link, insufficient protein solubilization during lysis.

Solutions: Check each batch of antibody by flow cytometry to ensure binding affinity and ensure cross-linking was effective. Confirm that all buffers are at the correct pH and were properly prepared. Optimize detergent type and concentration in lysis buffer and conditions for different cell types.

**Problem 2:** Peptide loss during concentration, resulting in low identification.

Possible issue: Sample lost during concentration.

Solution: Ensure that samples are not completely dried during centrifugal evaporation and that they are resuspended carefully. This avoids loss of material due to binding to the tube surface.

**Problem 3:** Low number of peptides identified.

Possible issue: The mass spectrometer is not performing optimally.

Solutions: Ensure that the mass spectrometer is functioning according to specifications by regularly tuning the instrument and running quality-control samples. In our laboratory, we use HeLa digested with Elastase as a QC standard to monitor instrument performance. Elastase is preferred over trypsin because it cleaves at more sites, providing a better proxy for immunopeptidomics workflows. For each instrument and method, we establish a benchmark. Deviations from these benchmarks indicate potential performance issues.
